# Supplementary material for: Outcome Measures and Biomarkers for Clinical Trials in Hereditary Spastic Paraplegia: A Scoping Review
Source: Genes (Basel). 2023 Sep 3;14(9):1756. doi: 10.3390/genes14091756 (PMC10530989; doi:10.3390/genes14091756)
Supplement: Supplementary file 1 [file genes-14-01756-s001.zip › Siow et al Supplementary Material S2.pdf]

Outcome measure/ Biomarker	Study	Classify patients vs controls		Indicate disease progression		Response to intervention		Correlation with other biomarker/outcome measure	
		Controls included	Outcome	Longitudinal study	Outcome	Intervention	Outcome	Biomarker	Significant correlation
**CROMs – HSP specific**									
SPRS	Ardolino 2021	No	N/A	No	N/A	Yes	No	No	N/A
	Brighente 2021	Yes	N/A	No	N/A	No	N/A	MEP amp or CMCT	No
	Chrestian 2016	No	N/A	No	N/A	No	N/A	Brain MRI	Yes
	Cubillos-Arcila 2022	Yes	N/A	Yes	Yes (but only in SPG4 group)	No	N/A	10MWT<br>6MWT and TUG	Yes<br>No
	deLima 2021	No	N/A	No	N/A	Yes	No	No	N/A
	DelaCasa-Fages 2019	No	N/A	No	No – descriptive paper	No	N/A	No	N/A
	Faber 2018	Yes	N/A	Yes	Yes	No	N/A	Disease duration MRI measures	Yes
	Gassner 2021	No	N/A	No	N/A	No	N/A	SF-12 PCS only, 10MWT, 2MWT, TUG.	Yes.
	Jordan 2021	No	N/A	No	N/A	No	N/A	CPCHILD	Yes
	Karle 2013	No	N/A	No	N/A	No	N/A	CMCT to the legs and arms.	Yes
	Kessler 2022	Yes	N/A	Yes	Yes (3/31 patients)	No	N/A	Serum Nfl	No
	Klimpe 2012	No	N/A	No	N/A	No	N/A	SF-36	Yes

	Lassman 2022	Yes	Yes	No	N/A	No	N/A	Gait analysis	Yes
	Lin 2021	Yes	N/A	No	N/A	No	N/A	CSF 27-OHC<br>CSF NfL	No
	Martinuzzi 2016	No	N/A	No	N/A	No	N/A	Age at onset and disease duration.<br>ENB2.<br>FIM<br>DTI - FA and MD.	Yes
	Montanaro 2020	Yes	N/A	Yes	No	No	N/A	VBM and DTI indices	No
	Musacchio 2018	No	N/A	Yes	Yes	No	N/A	No	N/A
	Navas-Sanchez 2021	Yes	N/A	No	N/A	No	N/A	Age<br>B/L thalamus volume	Yes
	Orsucci 2014	No	N/A	No	N/A	No	N/A	Age of onset.<br>Age at last evaluation.<br>SF36 score.	No (age)<br>Yes (SF-36)
	Paparella 2020	No	N/A	No	N/A	Yes	Yes	No	N/A
	Prestsaeter 2020	No	N/A	No	N/A	No	N/A	No	N/A
	Rattay 2022	Yes	Yes	No	N/A	No	N/A	CSF NfL	Yes
	Regensburger 2022	Yes	N/A	Yes	Yes (but only in patients with cHSP)	No	N/A	Mobile gait analysis parameters	Yes (weak)
	Rezende 2015	Yes	N/A	No	N/A	No	N/A	DTI indices	Yes (for TBSS but not

									tractography)
	Schols 2017	No	N/A	Yes	Yes	Yes	No	10MWT Time to climb 10 steps Serum 27-OHC	Yes
	Vavla 2019	No	N/A	Yes	N/A	No	N/A	RNFL thickness	No
	Wilke 2018	Yes	N/A	No	N/A	No	N/A	Serum Nfl	Yes
SPATAX-EUROSPA disability score	Chrestian 2016	No	N/A	No	No	No	N/A	No	N/A
	Dela-Casa Fages 2019	No	N/A	No	N/A	No	N/A	Variant type mtDNA	Yes No
	Kessler 2022	Yes	N/A	Yes	Yes (3/31 patients)	No	N/A	Serum Nfl	No
	Scheuer 2007	No	N/A	No	N/A	Yes	No	No	N/A
**CROMs – other conditions**									
SARA	Dela Casa Fages 2019	No	N/A	No	N/A	No	N/A	No	N/A
	Du Montcel 2008	Yes	Yes	No	N/A	No	N/A	No	N/A
	Du Montcel 2012	No	N/A	Yes	Yes	No	N/A	AMBUS	Yes
ALS rating scale revised (ALSFRS-R)	Simonini 2021	No	N/A	Yes	No	No	N/A	Serum and CSF crt-pNfH (for whole group including ALS)	Yes
	Zucchi 2018	Yes	N/A	Yes	Yes	No	N/A	CSF and Serum pNfH	Yes (with progression

									rate and total score)
Unified Huntington's Disease Rating Scale Part IV	Du Montcel 2008	Yes	N/A	No	N/A	No	N/A	CCFS	Yes
MS Impairment Scale	Scheuer 2007	No	N/A	No	N/A	Yes	No	No	N/A
**CROMs – Generic functional**									
Functional questionnaire score	Henderson 2019	No	N/A	No	N/A	Yes	No	No	N/A
Modified Rankin Scale	Zucchi 2018	Yes	N/A	Yes	N/A	No	N/A	Serum and CSF pNfH (whole group including ALS)	Yes (at last observation but not at diagnosis)
Disability Score (DIS)	Scheuer 2007	No	N/A	No	N/A	Yes	No	No	N/A
Functional Independence Measure (FIM)	Martinuzzi 2016	No	N/A	No	N/A	No	N/A	SPRS	Yes
**PerfOMs – motor function**									
Modified Ashworth Scale	Antczak 2019,	No	N/A	No	N/A	Yes	Yes (proximal not distal)	No	N/A
	Ardolino 2021	No	N/A	No	N/A	Yes	Yes	No	N/A
	Bastani 2021	No	N/A	No	N/A	Yes	Yes	No	N/A
	Bertolucci 2015	No	N/A	No	N/A	Yes	No	No	N/A

	Braschinsky 2009,	No	N/A	No	N/A	No	N/A	Active ROM (goniometer) Walking speed (10MWT)	Yes
	deLima 2021	No	N/A	No	N/A	Yes	Yes (hip adductors) No (triceps surae)	No	N/A
	Geva-Dayan 2010	No	N/A	No	N/A	Yes	Yes	No	N/A
	Kai 2014	No	N/A	Yes	Yes (immediately after treatment <12 months but not long term 12-24 months)	Yes	Yes	No	N/A
	Lith 2019	No	N/A	No	N/A	Yes	Yes	No	N/A
	Loureiro 2013	No	N/A	No	N/A	No	N/A	No	N/A
	Margetis 2014	No	N/A	No	N/A	Yes	Yes	No	N/A
	Martinuzzi 2016	No	N/A	No	N/A	No	N/A	No	N/A
	Paparella 2020	No	N/A	No	N/A	Yes	Yes	No	N/A
	Pointon 2022	No	N/A	No	N/A	Yes	Yes	No	N/A
	Simonini 2021	No	N/A	Yes	N/A	No	N/A	No	N/A
	Uygunoglo 2016	No	N/A	No	N/A	Yes	Yes	No	N/A
	Zhang 2014	Yes	N/A	No	N/A	Yes	N/A (not used as	No	N/A

							outcome measure)		
	Zucchi 2018,	Yes	N/A	Yes	No	No	N/A	Serum and CSF pNfH	Yes
10m walk time (10MWT), 6 MWT, 2 min WT, 5 min WT, 3-min endurance walk, 20m walk time	Antczak 2019,	No	N/A	No	N/A	Yes	No	No	N/A
	Ardolino 2021	No	N/A	No	N/A	Yes	No	No	N/A
	Bastani 2021	No	N/A	No	N/A	Yes	No	No	N/A
	Bertolucci 2015	No	N/A	No	N/A	Yes	Yes	No	N/A
	Braschinsky 2009	No	N/A	No	N/A	No	N/A	Active ROM as measured with Goniometer (hip abduction) MAS	Yes
	Cubillos-Arcila 2022	Yes	Yes	Yes	Yes (for max but not self-selected speed)	No	N/A	SPRS	Yes for 10MWT but no for 6MWT
	deLima 2021	No	N/A	No	N/A	Yes	No	No	N/A
	Gassner 2021	Yes	Yes	No	N/A	No	N/A	FES-I, SPRS, TUG, SF-12 (PCS only)	Yes
	Lith 2019	No	N/A	No	N/A	Yes	No	No	N/A
	Martinuzzi 2016	No	N/A	No	N/A	No	N/A	No	N/A
	Paparella 2020	No	N/A	No	N/A	Yes	Yes	No	N/A
	Pointon 2022	No	N/A	No	N/A	Yes	No	No	N/A
	Rattay 2022	Yes	No	No	N/A	No	N/A	No	N/A
	Uygunoglu 2016	No	N/A	No	N/A	Yes	Yes	No	N/A

Timed Up and Go test (TUG)	Antczak 2019	No	N/A	No	N/A	Yes	No	No	N/A
	Bertolucci 2015	No	N/A	No	N/A	Yes	No (close to significance)	No	N/A
	Cubillos-Arcila 2022	Yes	Yes	Yes	Yes for max but not self selected speed	No	N/A	SPRS	No
	Gassner 2021	Yes	Yes	No	N/A	No	N/A	FES-I<br>SPRS<br>SF-12	Yes<br>Yes<br>No
	Lith 2019	No	N/A	No	N/A	Yes	No	No	N/A
	Paparella 2020	No	N/A	No	N/A	Yes	Yes	No	N/A
	Regensburger 2022	Yes	N/A	Yes	Yes (for cHSP)	No	N/A	No	N/A
MRC muscle strength	deLima 2021	No	N/A	No	N/A	Yes	No	No	N/A
	Lith 2019	No	N/A	No	N/A	Yes	Yes (Hip adductors but not abductors)	No	N/A
	Loureiro 2013	No	N/A	No	N/A	No	N/A	No	N/A
	Martinuzzi 2016	No	N/A	No	N/A	No	N/A	No	N/A
Gross motor function measure (GMFM-66 or GMFM-88)	Geva-Dayan 2010	No	N/A	No	N/A	Yes	Yes	No	N/A
	Pointon 2022	No	N/A	No	N/A	Yes	No	No	N/A
Gross motor function classification score (GMFCS)	Mishra 2015	Yes	N/A	No	N/A	No	N/A	TSFA (DTI) in corticospinal tract	Yes
	Pointon 2022	No	N/A	No	N/A	Yes	No	No	N/A

Falls Efficacy Scale-International (FES-I)	Gassner 2021	Yes	Yes	No	N/A	No	N/A	SPRS<br>10MWT, 2MWT, TUG<br>SF-12	Yes.<br>No for total SF-12 (but yes for PCS)
	Regensburger 2022	Yes	N/A	Yes	N/A	No	N/A	Gait parameters (stride and stance time)	Yes
Berg balance scale	Bertolucci 2015	No	N/A	No	N/A	Yes	Yes	No	N/A
	Lith 2019	No	N/A	No	N/A	Yes	No	No	N/A
Composite cerebellar functional severity score (CCFSw)	Du Montcel 2008	Yes	Yes	No	N/A	No	N/A	UHDRS-IV<br>VAS (EQ5D)	Yes
	Du Montcel 2012	No	N/A	Yes	No	No	N/A	AMBUS	Yes
Nine hole pegboard test, click test, writing test, tapping test	DuMontcel 2008	Yes	No	No	N/A	No	N/A	No	N/A
	Uygunoglu 2016	No	N/A	No	N/A	Yes	Yes (left hand)<br>No (right hand)	No	N/A
Physiological Cost Index (increase in HR per metre walked, estimate of oxygen consumption)	Bertolucci 2015	No	N/A	No	N/A	Yes	No	No	N/A
	Schols 2017	Yes	N/A	Yes	N/A	Yes	No	No	N/A

Four-stage functional scale of motor disability (4SMD or 4FMS)	Orsucci 2014	No	N/A	No	N/A	No	N/A	SPRS, SF-36	Yes
	Jordan 2021	No	N/A	No	N/A	No	N/A	CPCHILD	Yes
Activities specific Balance Confidence scale (ABC)	Lith 2019	No	N/A	No	N/A	Yes	No	No	N/A
Modified version of Gillette Functional Assessment Questionnaire	Margetis 2014	No	N/A	No	N/A	Yes	Yes	No	N/A
Walking Handicap scale	Paparella 2020	No	N/A	No	N/A	Yes	Yes	No	N/A
Lower extremity subclass of Fugl-Meyer assessment	Bastani 2021	No	N/A	No	N/A	Yes	No	No	N/A
Ambulatory score (AMB)	Scheuer 2007	No	N/A	No	N/A	Yes	No	No	N/A
AMBUS (distance in metres walked in 5	DuMontcel 2012	No	N/A	Yes	N/A	No	N/A	SARA CCFSw	Yes

sec without help)									
Spasm frequency Scale	Kai 2014	No	N/A	Yes	Yes (post Rx)	Yes	Yes	No	N/A
Strength with microFET 2 hand-held dynanometer	Antczak 2019	No	N/A	No	N/A	Yes	Yes	No	N/A
Walking ability (landmarks of disability)	Klimpe 2012	No	N/A	No	N/A	No	N/A	SF-36	Yes
**PerfOMs – Cognition**									
Montreal Cognitive Assessment (MoCA)	Gassner 2021	Yes	N/A	No	N/A	No	N/A	No	N/A
	Rattay 2022	Yes	No	No	N/A	No	N/A	No	N/A
	Rattay 2019	Yes	No	No	N/A	No	N/A	SPRS	No
	Regensburger 2022	Yes	N/A	Yes	N/A	No	N/A	Gait stride parameters	Yes for coefficient of variation but not absolute values.
WAIS-R	Erichsen 2009	Yes	No	No	N/A	No	N/A	CVLT-T (multiple neuropsych tests performed)	Yes
	Musacchio 2018	No	N/A	Yes	Yes	No	N/A	No	N/A
	Martinuzzi 2016	No	N/A	No	N/A	No	N/A	No	N/A

MMSE	Navas-Sanchez 2021	Yes	N/A	No	N/A	No	N/A	No	N/A
	Lin 2021	Yes	N/A	No	N/A	No	N/A	CSF 27OHC, MMSE	No
Addenbrooke's Cognitive Examination Revised (ACE-R)	Faber 2018	Yes	N/A	Yes	N/A	No	N/A	SPRS	Yes
CANTAB Cognitive Assessment Software	Rattay 2019	Yes	No	No	N/A	No	N/A	No	N/A
VLMT, FWIT, TMT A/B, FAB, d2-R, RWT	Musacchio 2018	No	N/A	Yes	Yes	No	N/A	No	N/A
**PROMs – Quality of life**									
SF-36, SF-12, RAND 36-Item Health Survey	Bastani 2021	No	N/A	No	N/A	Yes	No	No	N/A
	Bertolucci 2015	No	N/A	No	N/A	Yes	Yes	No	N/A
	Braschinsky 2011	Yes (previously published values)	Yes	No	N/A	No	N/A	No	N/A
	Gassner 2021	Yes	Yes	No	N/A	No	N/A	FES-I, SPRS TUG, 10MWT, MWT	Yes Yes for PCS but not total
	Klimpe 2012	No	N/A	No	N/A	No	N/A	SPRS, Landmarks of disability	Yes
	Orsucci 2014	No	N/A	No	N/A	No	N/A	4SMD, SPRS	Yes

	Regensburger 2022	Yes	N/A	Yes	N/A	No	N/A	Gait parameters	Yes for PCS
EQ-5D	DuMontcel 2008	Yes	N/A	No	N/A	No	N/A	CCFS	Yes
	Rattay 2019	Yes	Yes	No	N/A	No	N/A	Maximum gait distance<br>BPI<br>BDI-V	Yes<br><br>No (RLS)
	Rattay 2022	Yes	No (pre SPG4)	No	N/A	No	N/A	No	N/A
Becks Depression Inventory (BDI-V)	Rattay 2022	Yes	No (pre SPG4)	No	N/A	No	N/A	No	N/A
	Rattay 2019	Yes	Yes	No	N/A	No	N/A	EQ5D	Yes
Visual analogue score (for QoL)	Paparella 2020	No	N/A	No	N/A	Yes	Yes	No	N/A
	Scheuer 2007	No	N/A	No	N/A	Yes	No	No	N/A
Patient Health Questionnair e (PHQ-9)	DuMontcel 2008	Yes	N/A	No	N/A	No	N/A	No	N/A
Modified Goal Attainment Scale (mGAS)	Pointon 2022	No	N/A	No	N/A	Yes	Yes	No	N/A
ICIQ-LUTSqol	Schneider 2019	No	N/A	No	N/A	No	N/A	ICIQ-SF, SCOPA-AUT	Yes
Cerebral Palsy QoL questionnair e (CPQoL)	Pointon 2022	No	N/A	No	N/A	Yes	Improved in 2/5 participants	No	N/A

CPCHILD questionnaire	Jordan 2021	No	N/A	No	N/A	No	N/A	4FMS	Yes
ZUNG depression score	Gassner 2021	Yes	Yes	No	N/A	No	N/A	No	N/A
ICIQ-SF	Schneider 2019	No	N/A	No	N/A	No	N/A	ICIQ-LUTSqoL	Yes
Hospital Anxiety and Depression Scale (HADS)	Bertolucci 2015	No	N/A	No	N/A	Yes	Yes	No	N/A
**PROMs – Other**									
Brief pain inventory	deLima 2021	No	N/A	No	N/A	Yes	No	No	N/A
	Rattay 2019	Yes	Yes	No	N/A	No	N/A	EQ5D	Yes
	Rattay 2022	Yes	No	No	N/A	No	N/A	No	N/A
Modified Fatigue Impact Scale (MFI)	deLima 2021	No	N/A	No	N/A	Yes	No	No	N/A
	Rattay 2019	Yes	Yes	No	N/A	No	N/A	EQ5D	Yes
Multidimensional fatigue inventory	Rattay 2022	Yes	No	No	N/A	No	N/A	No	N/A
SCOPA-AUT	Schneider 2019	No	N/A	No	N/A	No	N/A	ICIQ-LUTSQoL	Yes
Numeric rating scale for pain	Paparella 2020	No	N/A	No	N/A	Yes	Yes	No	N/A
**Biochemical/Lab studies**									
Serum and CSF neurofilament light chain	Kessler 2021	Yes	Yes (CSF NfL)	No	N/A	No	N/A	Age<br>Gender	Yes
	Kessler 2022	Yes	Yes (Serum NfL)	Yes	No	No	N/A	Age<br>SPRS	Yes<br>No

	Lassmann 2022	Yes	Yes	No	N/A	No	N/A	Gait parameter (foot angle and RoM)	Yes (serum NfL)<br>No (CSF NfL)
	Lin 2021	No (historical controls)	N/A (Yes compared to historical controls)	No	N/A	No	N/A	Spinal cord area, SPRS, MMSE<br>CSF 27-OHC	No<br><br>Yes (27-OHC)
	Rattay 2022	Yes	Serum - Yes for symptomatic SPG4, No for presymptomatic SPG4<br>CSF- yes for both	No	N/A	No	N/A	SPRS	Yes (CSF NfL)
	Wilke 2018	Yes	Yes	No	N/A	No	N/A	Disease progression (SPRS/disease duration)	Yes
Serum and CSF 24-OHC, 25-OHC and 27-OHC and 3b-CA	Lin 2021	No (previously reported healthy controls)	N/A (Yes compared to prev HC)	No	N/A	No	N/A	Spinal cord area, SPRS, MMSE<br>CSF NfL	No<br><br>Yes (Nfl)
	Prestsaeter 2020	No (reference range used)	N/A (Yes compared to reference range)	No	N/A	No	N/A	No	N/A
	Schols 2017	Yes	Yes for all except 24-OHC	Yes	N/A	Yes	Yes for serum 27-OHC, 25-OHC and 24 OHC	SPRS	Yes for 27-OHC only

							(No for CSF or 3b-CA)		
	Marelli 2018	Yes	Yes (plasma 27-OHC and 25-OHC)<br>No (24S-OHC)	Yes	No	Yes	Yes (plasma 27-OHC and 24S-OHC)<br>No (25-OHC)	No	N/A
Blood(plasma) and CSF amino acids	Coutelier 2015	Yes (historical controls)	Yes (ornithine, citrulline, arginine, proline)	No	N/A	No	N/A	No	N/A
	Growdon 1991	No	N/A	No	N/A	Yes	No (plasma and CSF glycine)	No	N/A
	Marelli 2020	No	N/A<br>Low citrulline compared to reference value	No	N/A	No	N/A	No	N/A
Lipidomics: fibroblast and plasma	Vaz 2019	Yes	Yes (plasma PC etherphospholipids)	No	N/A	No	N/A	No	N/A
Glycosylceramide profile	Kloth 2020	No	Increased compared to reference values	No	N/A	No	N/A	No	N/A
Neurofilament heavy chain: CSF and serum	Simonini 2021	No (but differentiated from ALS and UMNp)	N/A	No	N/A	No	N/A	Progression rate and total ALSFRS-R score (for whole group	Yes

								including ALS)	
	Zucchi 2018	Yes	Yes	Yes	N/A	No	N/A	ALSFRS-R total score and progression rate, Ashworth scale (for whole group including ALS)	Yes
ATG9A ratio	Ebrahimi-Fakhari 2021	Yes	Yes	No	N/A	No	N/A	No	N/A
CSF A beta 1-42, total tau, phosho tau	Rattay 2022	Yes	No	No	N/A	No	N/A	No	N/A
mtDNA levels; Muscle biopsy	DelaCasa-Fages 2019	Yes	Yes	No	N/A	No	N/A	Age of onset, degree of spasticity, disability scale	No
Cell morphometry	Wali 2021	Yes	Yes	No	N/A	Yes	Yes (SPAST only)	No	N/A
Scanning electron microscopy of hair shafts	Rattay 2019	Yes	Yes	No	N/A	No	N/A	No	N/A
**Imaging**									
MRI brain and spine,	Chrestian 2016,	No	N/A	No	N/A	No	N/A	SPRS	Yes

volumetry with neural network analysis, automated segmentation of subcortical structures	DelaCasa-Fages 2019	No	N/A	No	N/A	No	N/A	No	N/A
	Ebrahimi-Fakhari 2021	Yes	N/A	No	N/A	No	N/A		
	Faber 2018	Yes	Yes (cortical thickness, deep gray matter volume, spinal cord area)	Yes	N/A	No	N/A	ACE-R, SPRS, disease duration	Yes
	Lin 2021	Yes	Yes (spinal cord area) No (cerebral atrophy)	No	N/A	No	N/A	CSF Nf, 27-OHC	No
	Marelli 2018	Yes	N/A	Yes	N/A	Yes	N/A	No	N/A
	Martinuzzi 2016	No	N/A	No	N/A	No	N/A	No	N/A
	Montanaro 2020	Yes	Yes (right prefrontal cortx & B/L thalami vol)	Yes	No	No	N/A	SPRS, disease duration	No
	Navas-Sanchez 2021	Yes	Yes (thalamic atrophy)	No	N/A	No	N/A	SPRS Disease duration	Yes
	Orsucci 2014	No	N/A	No	N/A	No	N/A	No	N/A
	Prestsaeter 2020	No	N/A	No	N/A	No	N/A	No	N/A
	Rattay 2019	No	N/A	No	N/A	No	N/A	No	N/A
	Rattay 2022	Yes	Yes	No	N/A	No	N/A	No	N/A
	Rezende 2015	Yes	No (cortical thickness)	No	N/A	No	N/A	DTI (FA R CST, spinal cord area)	Yes

			Yes (spinal cord atrophy)						
	Sarret 2016	No	N/A	Yes	Yes (atrophy score, cerebellar volume)	No	N/A	No	N/A
	Schuurs-Hoeijmakers 2012	No	N/A	No	N/A	No	N/A	No	N/A
	Simonini 2021	No	N/A	Yes	N/A	No	N/A	No	N/A
DTI	Aghakhanyan 2014	Yes	Yes(FA, MD, RD)	No	N/A	No	N/A	No	N/A
	Faber 2018	Yes	Yes (FA, MD, AD, RD)	Yes	N/A	No	N/A	ACE-R	Yes
	Martinuzzi 2016	Yes	Yes	No	N/A	No	N/A	SPRS	Yes
	Mishra 2015	Yes	Yes (lower FA)	No	N/A	No	N/A	GMFCS	Yes for DTFA in 1 section of CST
	Montanaro 2020	Yes	Yes	Yes	Yes	No	N/A	SPRS	No
	Rezende 2015	Yes	Yes	No	N/A	No	N/A	SPRS (TBSS but not tractography)<br>Spinal cord area	Yes
	Unrath 2010	Yes	Yes	No	N/A	No	N/A	No	N/A
MR spectroscopy	Erichsen 2009	Yes	Yes (Cho/Cr ratio)	No	N/A	No	N/A	CVLT	Yes (Cho/Cr ratio)
	Martinuzzi 2016	Yes	No	No	N/A	No	N/A	No	N/A

	Montanaro 2020	Yes	Yes	Yes	No	No	N/A	No	N/A
	Schuurs-Hoeijmakers 2012	No	N/A (abnormal lipid peak in basal ganglia and thalamus in 5/5 patients)	No	N/A	No	N/A	No	N/A
Ioflupane SPECT	DelaCasa-Fages 2019	No	N/A (abnormal in 2/3 patients)	No	N/A	No	N/A	No	N/A
**Neurophysiology**									
MEPs	Antczak 2019	No	N/A	No	N/A	Yes	No	Strength post-rTMS	Lower MT correlated with bigger improvement in strength with rTMS
	Ardolino 2021	No	N/A	No	N/A	Yes	No	No	N/A
	Brighente 2021	Yes	Yes	No	N/A	No	N/A	Age, disease duration, SPRS	No
	Karle 2013	No (used normal values)	N/A	No	N/A	No	N/A	SPRS	Yes (UL and LL CMCT)
	Lassmann 2022	Yes	No	No	N/A	No	N/A	Gait parameters (leg RoM, foot angle)	Yes
	Martinuzzi 2016	No	N/A	No	N/A	No	N/A	No	N/A

	Musacchio 2018	No	N/A	Yes	N/A	No	N/A	No	N/A
	Rattay 2019	No	N/A	No	N/A	No	N/A	No	N/A
	Scheuer 2007	No	N/A	No	N/A	Yes	No	No	N/A
	Simonini 2021	No	N/A	Yes	N/A	No	N/A	No	N/A
NCS/EMG	Ardolino 2021 (H-reflex, F-waves)	No	N/A	No	N/A	Yes	No	No	N/A
	DelaCasa-Fages 2019	No	N/A	No	N/A	No	N/A	No	N/A
	Faber 2018	Yes	N/A	Yes	N/A	No	N/A	No	N/A
	Karle 2013	No	N/A	No	N/A	No	N/A	CMCT	Yes (NCV and F wave latency)
	Martinuzzi 2016	No	N/A	No	N/A	No	N/A	No	N/A
	Musacchio 2018	No	N/A	Yes	N/A	No	N/A	No	N/A
	Prestsaeter 2020	No	N/A	No	N/A	No	N/A	No	N/A
	Rattay 2019	No	N/A	No	N/A	No	N/A	No	N/A
	Rattay 2022	Yes	No	No	N/A	No	N/A	No	N/A
SSEP	Brighente 2021	Yes	Yes (LL only)	No	N/A	No	N/A	Age, disease duration<br>SPRS	Yes<br><br>No
	Karle 2013	No	N/A	No	N/A	No	N/A	No	N/A
	Martinuzzi 2016	No	N/A	No	N/A	No	N/A	No	N/A
	Prestsaeter 2020	No	N/A	No	N/A	No	N/A	No	N/A
	Rattay 2019	No	N/A	No	N/A	No	N/A	No	N/A

VEP	Prestsaeter 2020	No	N/A	No	N/A	No	N/A	No	N/A
	Rattay 2019	No	N/A	No	N/A	No	N/A	No	N/A
BAEP	Prestsaeter 2020	No	N/A	No	N/A	No	N/A	No	N/A
**Other biomarkers**									
Laboratory and mobile gait analysis	Adair 2016	No (normal values)	Yes (abnormal Gait Progile Score and Gait variable scores)	No	N/A	No	N/A	No	N/A
	Bonnefoy-Mazure 2013	Yes	Yes (normalized gait velocity, lower limb, thorax, pelvis and spine kinematics)	No	N/A	No	N/A	No	N/A
	Henderson 2019	No	N/A	No	N/A	Yes	No	No	N/A
	Lith 2019	No	N/A	No	N/A	Yes	Yes (comfortable gait speed)<br>No (Gait width, maximal gait speed)	No	N/A
	Lassman 2022	Yes	Yes	No	N/A	No	N/A	SPRS, serum NfL, MEP CMCT	Yes<br>No (CSF NfL)
	Pointon 2022	No	N/A	No	N/A	Yes	No	No	N/A

	Regensburger 2022	Yes	Yes	Yes	Yes for stride time, stance time and swing duration in cHSP subgroup.	No	N/A	SPRS, SF-12 PCS, FES-I	Yes
	Zhang 2014	Yes (historical)	Generally no but yes for some parameters (e.g. abnormal hip extension, increased hip int rotation)	No	N/A	Yes	Yes for walking speed, hip rot, knee rot, but no for cadence, step length/time, hip, knee, ankle F/E, Abd/Ad	No	N/A
SD-OCT Retinal nerve fibre layer	Klebe 2012	No	N/A (optic atrophy present in 10/10 patients examined)	No	N/A	No	N/A	No	N/A
	Musacchio 2018	No	N/A	Yes	N/A	No	N/A	No	N/A
	Prestsaeter 2020	No	N/A	No	N/A	No	N/A	No	N/A
	Vavla 2019	No (historical normative values)	Statistically significant reduction of RNFL thickness in	Yes (subgroup of patients)	No (avg F/U 10.7 months)	No	N/A	SPRS	No

			SUP, INF, NAS)						
Video-oculography and rotational chair testing	Milenkovic 2019	Yes	Yes (mean peak velocity vertical saccades)	No	N/A	No	N/A	No	N/A
RoM using goniometer	Braschinsky 2009	No	N/A	No	N/A	No	N/A	10MWT MAS	Yes
	Lith 2019	No	N/A	No	N/A	Yes	Yes	No	N/A
Instrumented dynamic balance assessment	Lith 2019	No	N/A	No	N/A	Yes	Yes (known direction) No (unknown direction)	No	N/A
Urodynamic assessment	Joussain 2019	No	N/A (majority had neurogenic detrusor overactivity)	No	N/A	No	N/A	No	N/A
Video supported posturography	Musacchio 2018	No	N/A	Yes	N/A	No	N/A	No	N/A
Protocol for Evaluation of Acquired Speech Disorders (PADAF)	Olchik 2021	Yes	Yes (abnormal resonance, phonation, breathing, articulation)	No	N/A	No	N/A	No	No
