# Supplementary material for: Outcome Measures and Biomarkers for Clinical Trials in Hereditary Spastic Paraplegia: A Scoping Review
Source: Genes (Basel). 2023 Sep 3;14(9):1756. doi: 10.3390/genes14091756 (PMC10530989; doi:10.3390/genes14091756)
Supplement: Supplementary file 1 [file genes-14-01756-s001.zip › Siow et al Supplementary Material S3.pdf]

Supplementary Material S3

Table S3A: Comparison of HSP Randomized Controlled Trials

	Growdon 1991	Scheuer 2007	Schols 2017	Antczak 2019	Ardolino 2021	Bastani 2021	deLima 2021	Van de Venis 2023
Aim	To assess the clinical effect of L-threonine in patients with HSP.	To investigate the effects of gabapentin on clinical disability and motor cortical excitability in SPG4-linked hereditary spastic paraplegia patients.	To perform a detailed clinical and biochemical analysis in 34 genetically confirmed SPG5 cases from 28 families, study dose-dependent neurotoxicity of oxysterols in human cortical neurons and perform a randomized placebo-controlled double blind interventional trial targeting oxysterol accumulation in serum of SPG5 patients.<br>2 phase: cross sectional study and randomized controlled trial	To investigate the therapeutic potential of bilateral, high-frequency repetitive TMS over primary motor areas for muscles of lower extremities in various forms of HSP (pure, complicated, AMN). RCT, crossover design. First study using rTMS for therapeutic purposes in HSP.	Double-blind, randomised crossover and sham-controlled study to evaluate the effects of tsDCS on spasticity in patients with HSP	To study the effectiveness of rTMS as palliative measure for lower limb spasticity, function and gait improvement in patients with HSP	To evaluate the efficacy and safety of BoNT-A in the treatment of lower limb spasticity in patients with HSP.	To assess efficacy of 5-week gait adaptability training in people with pure HSP
Number of participants	18 (8 declined lumbar puncture)	10	14	15 recruited (1 drop out)	11	8	55 enrolled (6 dropped out)	36
Genotype	Not specified	SPG4	SPG5	1 SPG3A<br>1 SPG7<br>3 AMN<br>3 AMN carrier<br>7 unknown (5 pure, 2 complex)	8 SPG4<br>1 SPG3A<br>1 SPG7<br>1 SPG15	Not specified	21 SPG4<br>4 SPG8<br>2 SPG3A<br>2 SPG11<br>2 SPG33<br>3 SPG72<br>1 SPG6<br>1 SPG7<br>1 SPG15<br>1 SPG28<br>1 SPG48<br>1 PLA2G6	Unknown

							1 X-linked adrenoleukodystrophy<br>1 autosomal-recessive spastic ataxia of Charlevoix-Saguenay<br>13 unknown	
Inclusion Criteria	Clinical diagnosis of HSP	Clinically affected. Genetic linkage to SPG4. Age 18-68yo.	Clinical diagnosis of HSP. Genetically confirmed SPG5. >10 years old.	Age >18 years old. Diagnosis of HSP and AMN confirmed by genetic testing or family history or by exclusion.<br>Able to walk 10 meters with or without crutches.	Diagnosis of HSP	Fink criteria (definitely or probably affected with HSP).<br>Other possible causes ruled out with CNS MRI, HTLV1 Ab titre, VDRL, vitamin B12, plasma VLCFAs, EMG, NCS.	Diagnosis of HSP based on either genetic testing or family history along with clinical features. 18-80 years of age. Able to walk at least 14m without stopping (assistive devices permitted).	Pure HSP diagnosed by movement disorder specialist, 18-70 years old, able to walk barefoot on level ground without a walking aid (orthopedic devices allowed)
Intervention	L-threonine: Patients received L-threonine at 1.5g TDS (n=7) or 2g TDS (n=11) for 2 weeks, 2-week washout period, then 2 week placebo. 2 groups receiving L-threonine>placebo or placebo>L-threonine.	Gabapentin commenced at 2400mg daily, gradually titrated to 4000mg over 10 days. 2 months treatment with gabapentin, 10 days washout, then 1 month drug free interval, 2 months placebo. Cross over design: 2 groups of placebo>gabapentin and gabapentin>placebo.	Atorvastatin 40mg/day for 9 weeks (n=7) or placebo for 9 weeks (n=7).	Repetitive transcranial magnetic stimulation (rTMS): 5 stimulating sessions, 1 a day during consecutive working days or sham rTMS. Crossover design, 8 patients received active treatment first.	Spinal direct current stimulation (tsDCS): 20 minute session twice a day 5 days a week with programmable stimulator or sham tsDCS. At least 3 months apart. Cross over design: 2 groups of tsDCS>sham and sham>tsDCS.	Repetitive transcranial magnetic stimulation (rTMS): 5 sessions rTMS on 5 consecutive days in a week (n=4) and sham rTMS (n=4).	Botulinum toxin type A (BoNT-A):<br>Single treatment with BoNT-A or placebo(0.9% sodium chloride solution) with 8wk follow up. Crossover with 24-28 weeks washout. Total BoNT-A dose 400 units (100 units in each adductor magnus and 100 units in each triceps surae).	Gait adaptability training on treadmill with augmented reality: 60 minutes twice a week for 5 weeks (Total 10 hours). Cross-over design: Participants randomized to intervention group (n=18) or waiting list control group (n=18). Following assessment after first 5 week period, the intervention group enters follow-up period and control groups starts 5-week of gait adaptability training.
Duration of intervention	2 weeks	2 months	9 weeks	5 days	5 days	5 days	8 weeks	5 weeks

Duration of intervention	2 weeks	2 months	9 weeks	5 days	5 days	5 days	8 weeks	5 weeks
Primary outcome measure	Disability score based on: Leg strength, leg tone, leg deep tendon reflexes, clonus, walking, hopping on right and left foot, running. Physician overall clinical assessment. Patient self-evaluation of response.	Primary vs Secondary not defined.<br>Clinical measures: MS Impairment Scale, SPATAX diagnostic form, lower limb patellar reflexes, muscle strength, muscle tone, disability score, ambulatory scoring (number of meters travelled in 5 sec without aid).<br>Self-reported parameters: severity of sphincter disturbances, Visual Analogue Scale score of quality of life.<br>Paired-transcranial magnetic stimulation (P-TMS).<br>Blood gabapentin levels.	Change of 27-OHC in serum after 9 weeks.	10-meter walk test (10MWT)	Primary vs Secondary not defined.<br>Ashworth scale, 5-minute walking test (n=9), motor evoked potentials (resting motor threshold, onset latency, AUC of motor response), H-reflex tibial nerve, F-waves abductor hallucis, SPRS (n=9)	Modified Ashworth Scale.	10-meter walk test (10MWT) to calculate maximal gait velocity.	Gait adaptability assessed with obstacle subtask of E-FAP
Secondary outcome measures	Amino acid analysis in blood and CSF.		Change of 27-OHC in CSF.<br>Reduction of 24-OHC, 25-OHC and 3β-CA levels in serum and CSF.<br>Serum bile acids.<br>SPRS, 3-min endurance walk, physical cost index.	MEP amplitude, CMCT, CSP.<br>Timed up and go test (TUG), strength measured with microFET 2 handheld dynamometer, modified Ashworth scale		Lower extremity subclass of Fugl-Meyer assessment.<br>10 meter walk test.<br>SF-36 Persian translation.	SPRS<br>Change in modified Ashworth scale and MRC for adductor and triceps surae.<br>Brief pain inventory.<br>Modified Fatigue Impact Scale.<br>Subjective perception of symptoms, or benefit, or gait improvement	Mini Balance Evaluation Test, Activities-specific Balance Confidence scale, Walking Adaptability Ladder Test (WALT), 10 meter Walk Test (10MWT), 3-D gait analysis (Vicon Motion Systems)

Results	**Statistically significant improvement in disability scores** with L-threonine compared to placebo.<br>Physician's overall clinical assessment and patients' self-evaluation of response demonstrated no significant difference between threonine and placebo.<br>L-threonine increased the mean blood and CSF threonine levels, no change in mean glycine levels in plasma or CSF with L-threonine vs placebo.<br>Overall, L-threonine did not produce clinically valuable improvement in motor unction although there was improvement in mean spasticity rating scores.	No statistical differences between gabapentin and placebo groups in median scores of self-reported and clinical ratings.<br>Blood showed increase in S-gabapentin in all patients during gabapentin treatment.<br>No evidence of altered intracortical excitability (compared average amplitude ratios) with P-TMS in both groups.	**Atorvastatin significantly reduced serum 27-OHC compared to placebo.**<br>Serum cholesterol lowered by 40% in atorvastatin but not placebo group.<br>Atorvastatin but not placebo reduced serum 24S-OHC and 25-OHC.<br>3β-CA levels showed large variability in both groups with no significant difference between groups.<br>No significant difference in changes of CSF 24S-OHC, 27-OHC or 3β-CA in both groups.<br>No effect of atorvastatin on SPRS or 3-min endurance walk or physical cost index.	No effect of rTMS in 10MWT and TUG.<br>Strength of proximal and distal muscles of lower extremities increased, and spasticity of proximal muscles decreased after rTMS.<br>Big range of motor threshold values, change of strength showed inverse correlation with mean MT, change in spasticity showed no significant correlation.	**Ashworth scale for lower limbs improved in anodal compared to sham group**, in particular up to 2 months following end of stimulation.<br>Significant improvement in hip flexion and knee extension.<br>No significant change in 5MWT, MEP variables, H-reflex, F-waves and SPRS between groups.	**rTMS had lower MAS scores at end of rTMS and end of follow up compared with sham group**.<br>No difference between both groups in Lower extremity subclass of Fugl-Meyer assessment, 10MWT and SF-36.	No change in maximal gait velocity (10MWT), SPRS, MRC(strength), BPI, MFIS or subjective perception between treatment and placebo groups.<br>Reduction in adductor muscle tone (MAS) in treatment compared to placebo but no significant change in triceps surae muscle.	Time required to perform E-FAP obstacle subtask did not decrease more in intervention compared to control group.<br>Non-significant results found for secondary outcomes except for single run of WALT.<br>5-weeks of gait adaptability training does not lead to greater improvement of gait adaptability compared to usual care.
